# Supplementary figures and images for: The Molecular Mechanism of the TEAD1 Gene and miR-410-5p Affect Embryonic Skeletal Muscle Development: A miRNA-Mediated ceRNA Network Analysis
Source: Cells. 2023 Mar 20;12(6):943. doi: 10.3390/cells12060943 (PMC10047409; doi:10.3390/cells12060943)

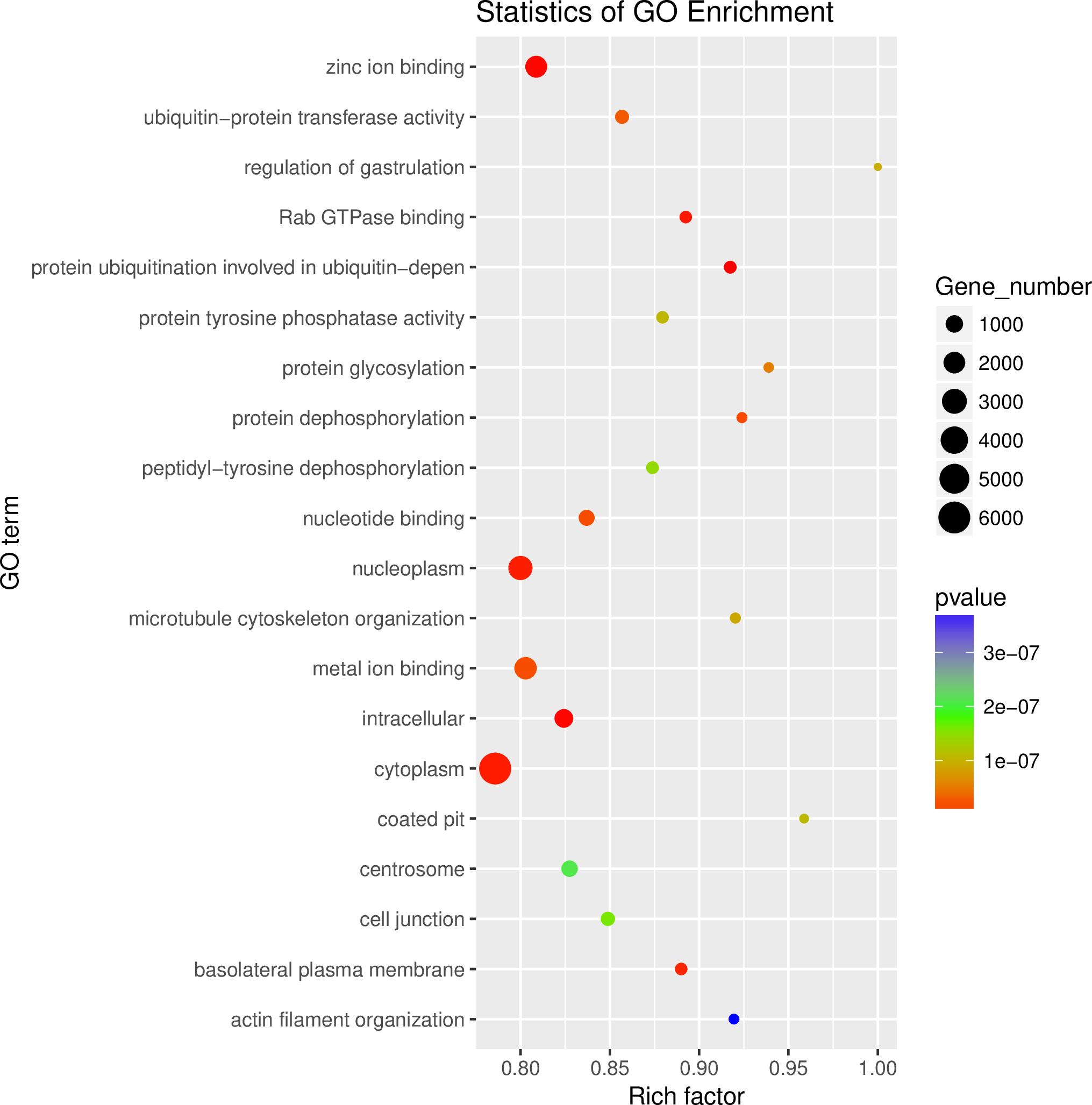

Supplement: Supplementary file 1 [file cells-12-00943-s001.zip › Figure S1 (A).tif]

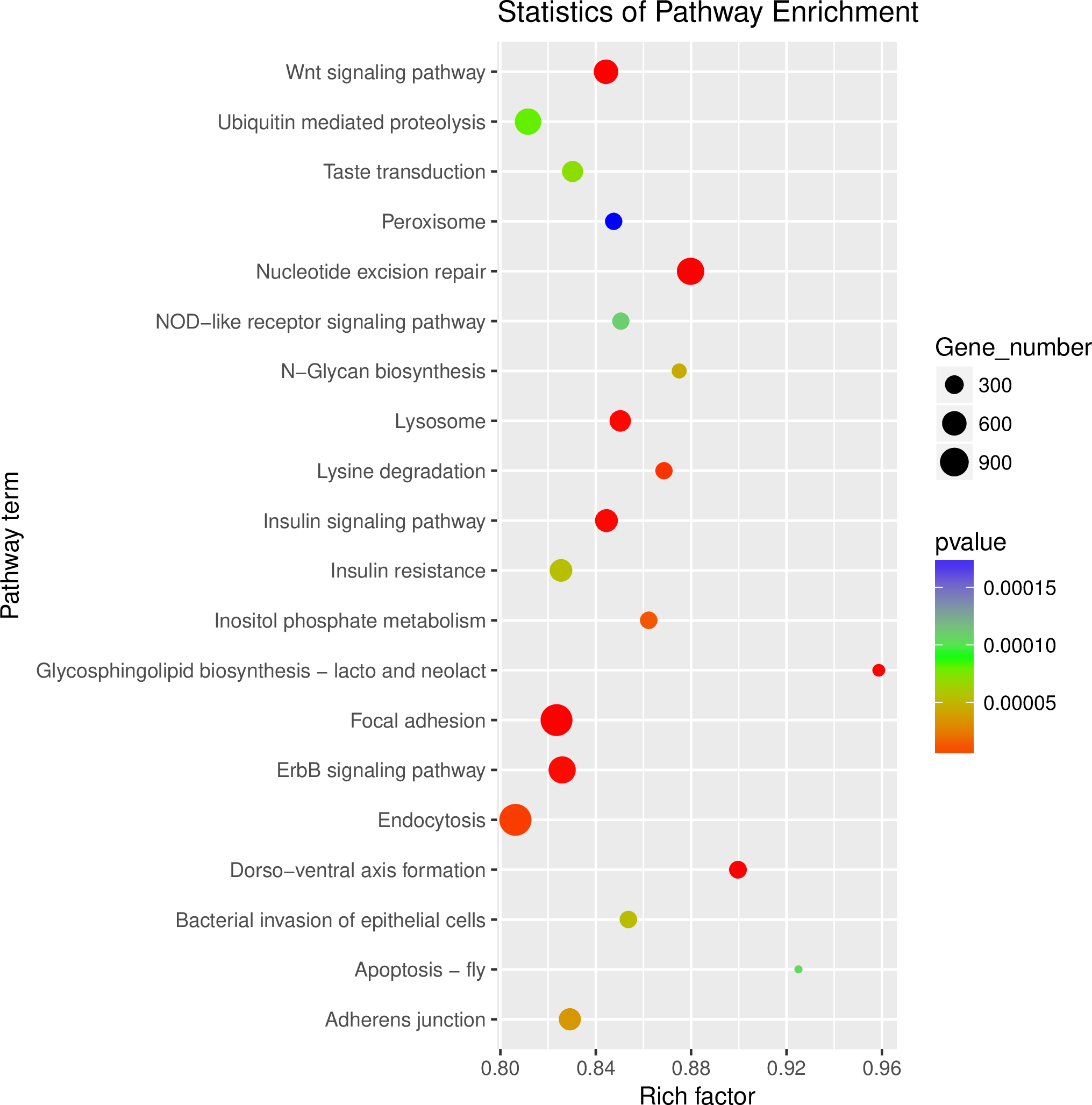

Supplement: Supplementary file 1 [file cells-12-00943-s001.zip › Figure S1 (B).tif]

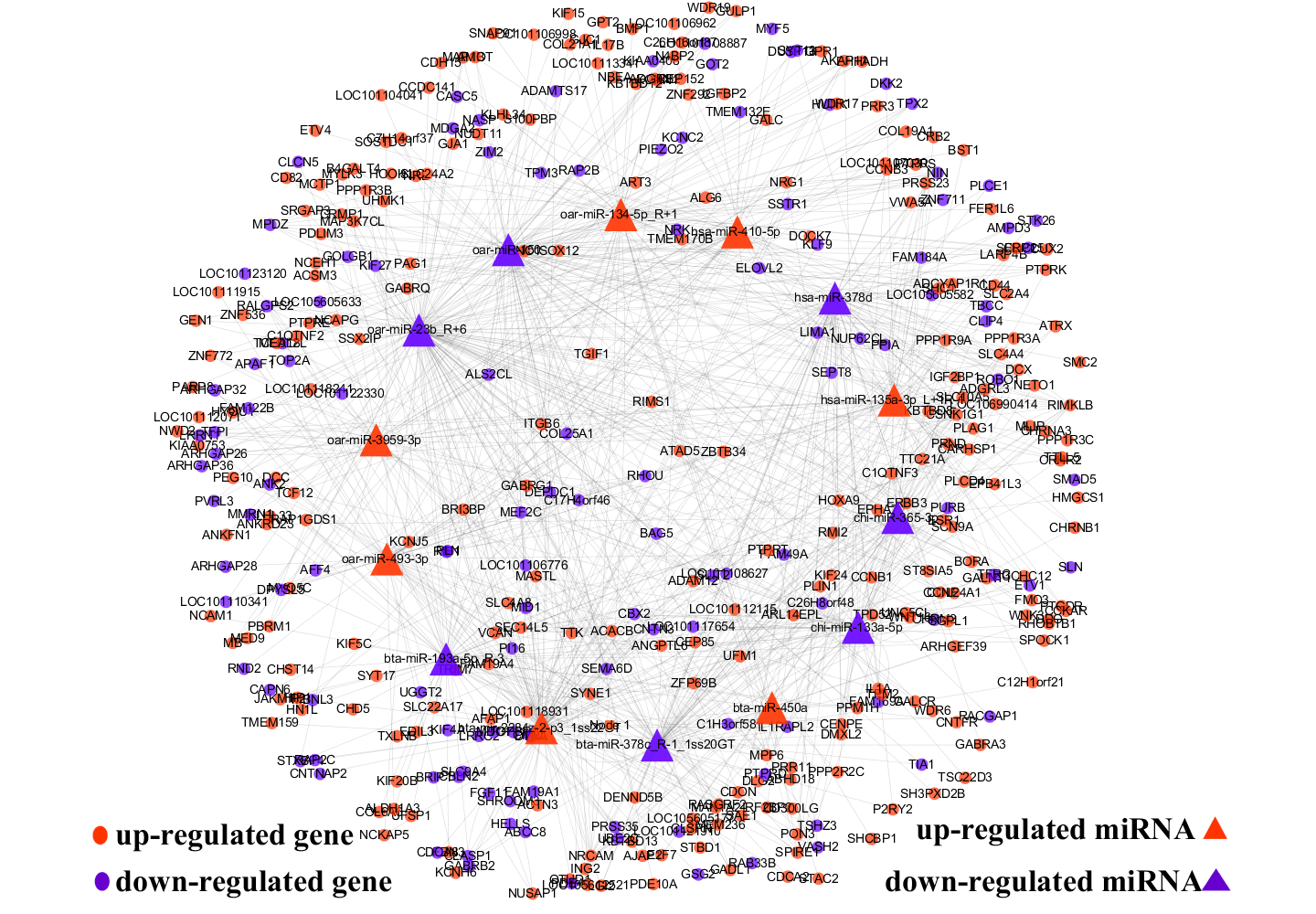

Supplement: Supplementary file 1 [file cells-12-00943-s001.zip › Figure S2.tif]

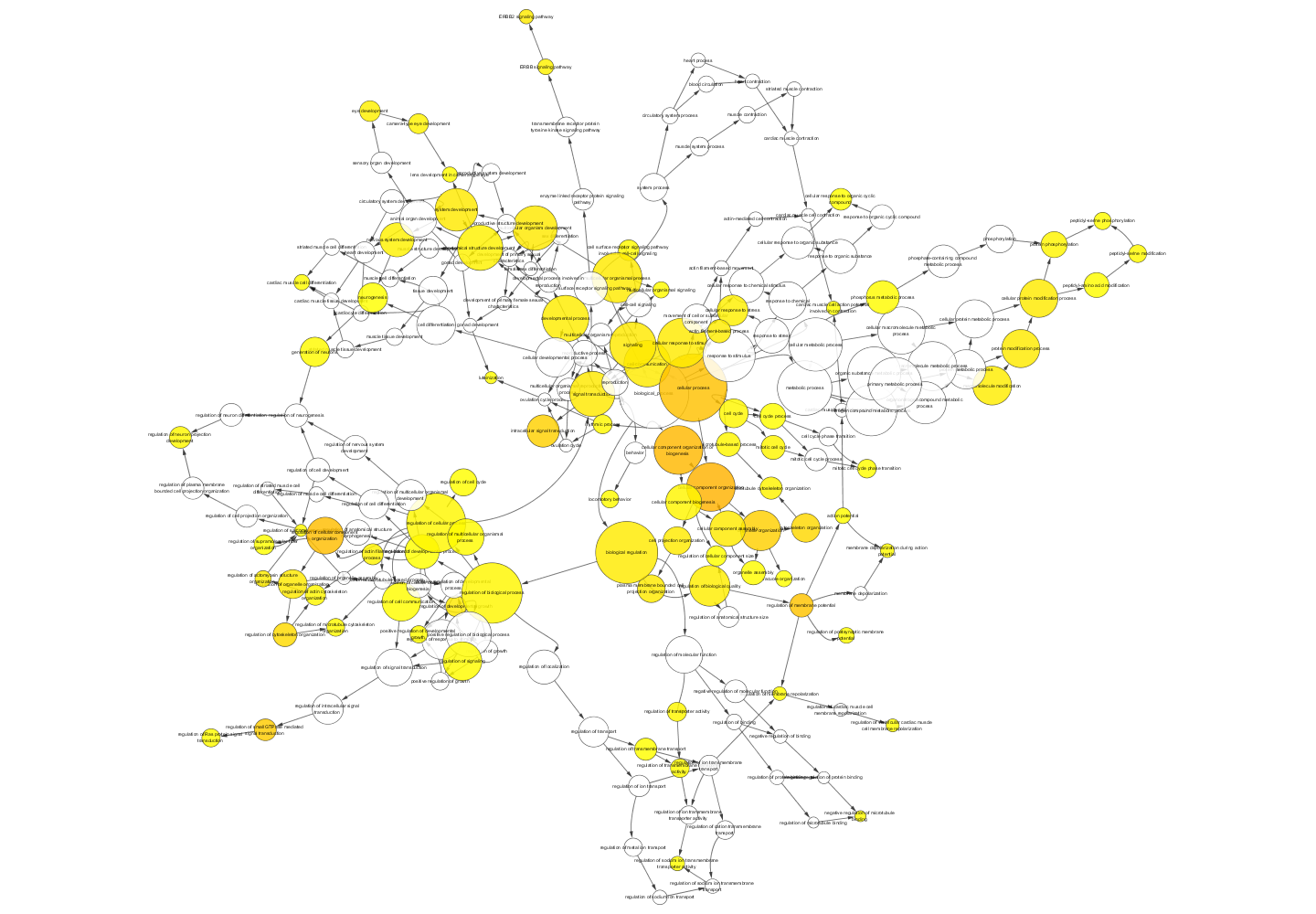

Supplement: Supplementary file 1 [file cells-12-00943-s001.zip › Figure S3 (A).tif]

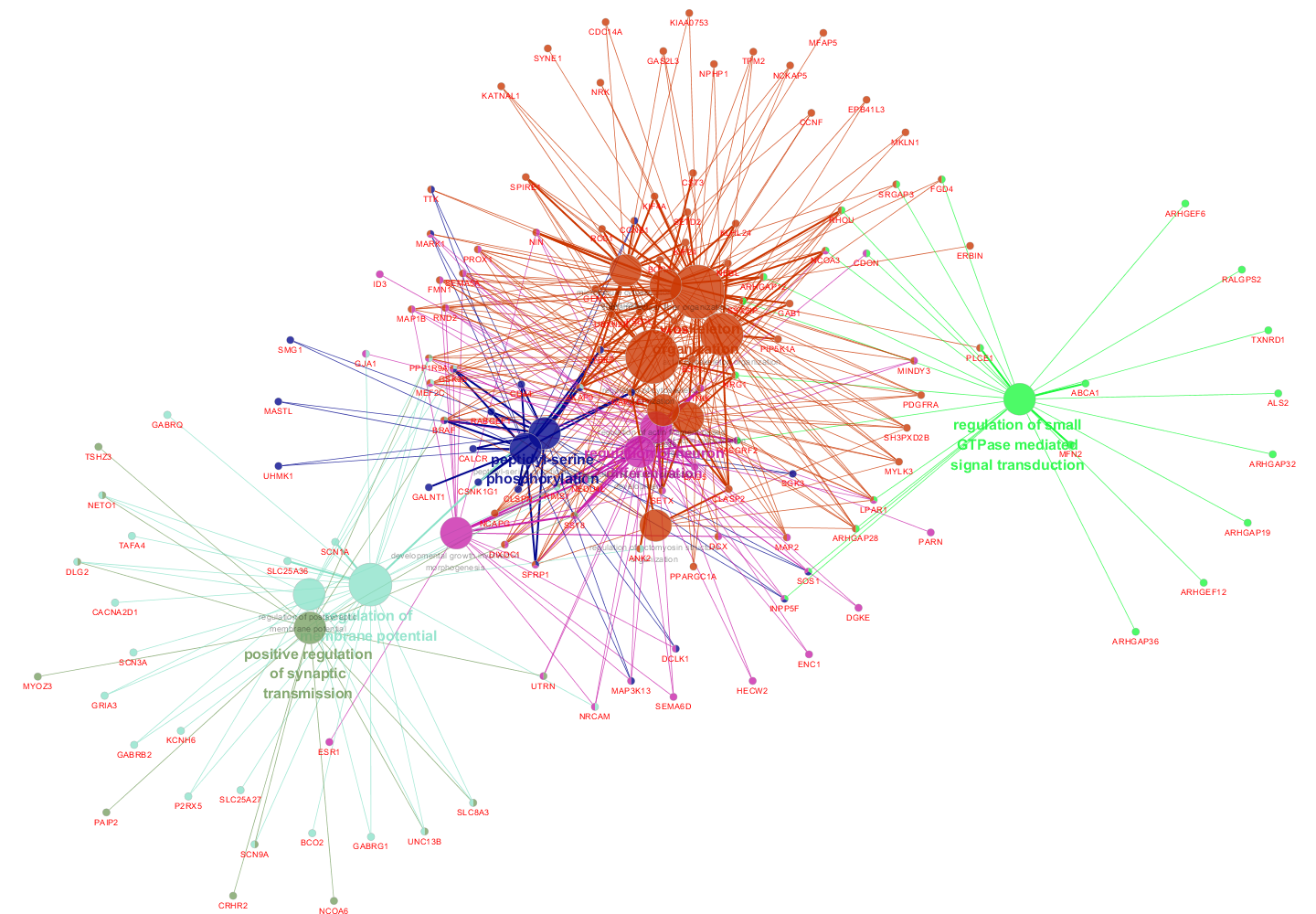

Supplement: Supplementary file 1 [file cells-12-00943-s001.zip › Figure S3 (B).tif]

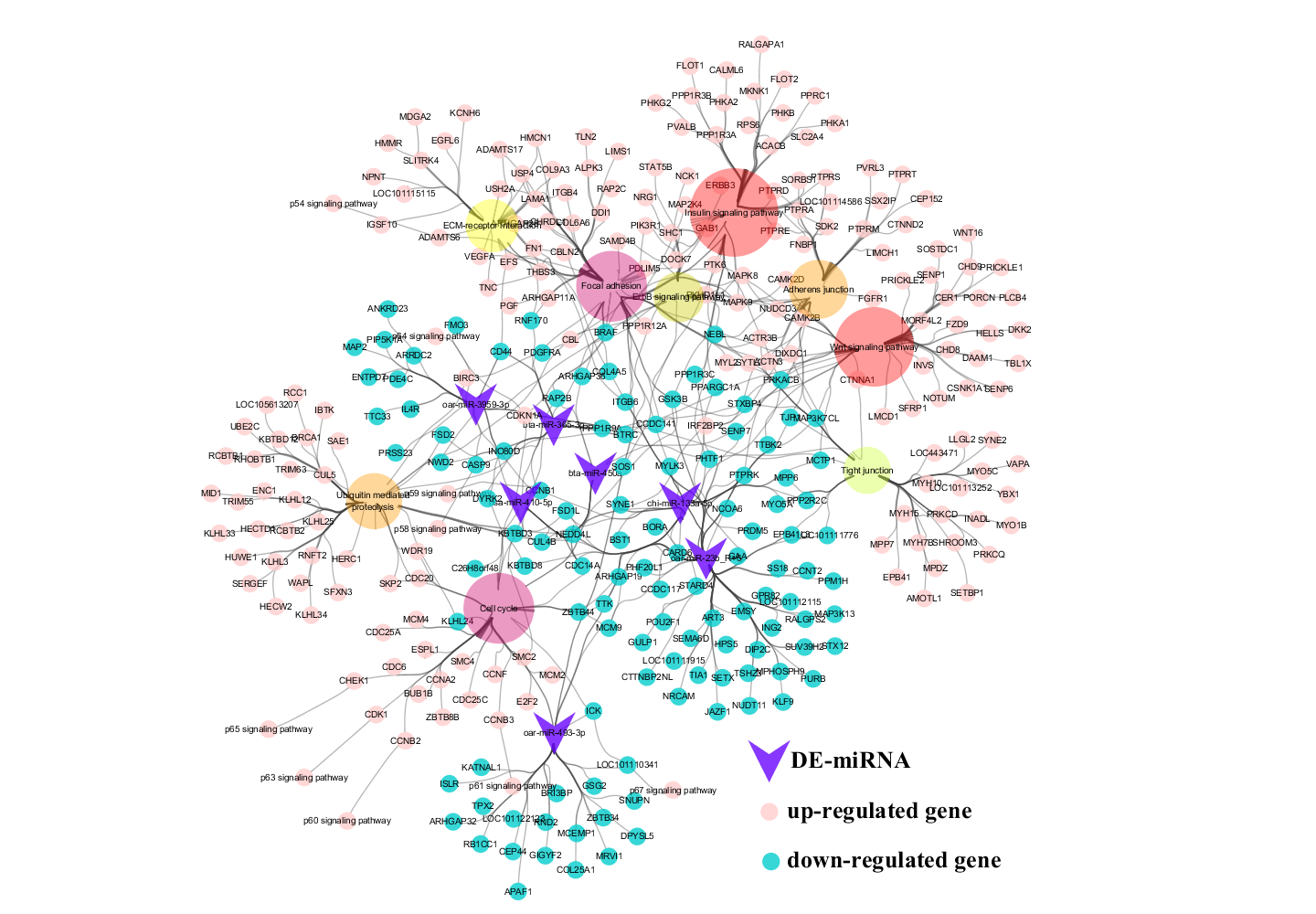

Supplement: Supplementary file 1 [file cells-12-00943-s001.zip › Figure S4.tif]
